# Opposing BMP and Wnt signaling drive astrocyte-like phenotypic transition of oligodendrocyte precursor cells at the expense of remyelination in a mouse model of demyelination

**DOI:** 10.3389/fncel.2026.1869788

**Published:** 2026-10-28

**Authors:** Shouyu Wang, Zhonghao Wu, Xiaoying Chen, Jianqin Niu, Yuxin Wang

**Affiliations:** 1 Biomedical Analysis Centre, Third Military Medical University, Chongqing, China; 2 Department of Histology and Embryology, Third Military Medical University, Chongqing, China; 3 Chongqing Key Laboratory of Neurobiology, Chongqing, China; 4 State Key Laboratory of Trauma and Chemical Poisoning, Chongqing, China; 5 Army 953 Hospital, Shigatse Branch of Xinqiao Hospital, Third Military Medical University, Shigatse, China; 6 Department of Neurosurgery, Xinqiao Hospital, Third Military Medical University, Chongqing, China

**Keywords:** astrocyte, BMP, oligodendrocyte precursor cell, remyelination, transformation, Wnt

## Abstract

Oligodendrocyte precursor cells (OPCs) are a widely distributed glial population in the adult central nervous system that retain the ability to differentiate into mature oligodendrocytes (OLs), or under pathological conditions, adopt alternative phenotypic transition. The post-injury phenotype of OPCs critically determines remyelination efficiency, yet the molecular cues controlling OPC transformation and their impact on myelin repair remain poorly understood. Here, using PDGFRα-CreER; Rosa-YFP transgenic mice for lineage tracing and a Lysophosphatidylcholine-induced focal demyelination model, we show that OPCs acquire astrocytic characteristics within lesions, accompanied by opposing activation of bone morphogenic protein (BMP) and Wnt signaling. Specifically, transforming OPCs exhibit marked downregulation of β-catenin, phosphorylated glycogen synthase kinase-3 beta at serine 9 (p-Ser9-GSK-3β) and Smad7, together with upregulation of phosphorylated Smad1/5 (p-Smad1/5), reflecting concurrent BMP activation and Wnt inhibition. Although pharmacological activation of BMP or inhibition of Wnt alone had no effect, suggesting an antagonistic interplay, counteracting endogenous BMP upregulation or Wnt downregulation effectively governed the balance between OPC differentiation and transformation. Notably, combined inhibition of BMP and Wnt significantly reduced astrocyte accumulation in lesions and expanded the MBP^+^ remyelinated area, indicating that blocking both signals diverts OPCs from astrocyte-like phenotypic transition and promotes robust remyelination. Together, these findings reveal that opposing activation of BMP and Wnt drives the astrocyte-like phenotypic transition of OPCs and impairs myelin repair, positioning this signaling axis as a promising therapeutic target to enhance remyelination in multiple sclerosis.

## 1 Introduction

Multiple sclerosis (MS) is a chronic inflammatory demyelinating neurodegenerative disease and a leading cause of non-traumatic disability in young adults (Jakimovski et al., 2024). Current immunotherapies primarily target inflammation in the central nervous system (CNS) and exert disease-modifying effects on relapses, clinical disability, and neuroimaging outcomes (Baecher-Allan et al., 2018; Konen et al., 2023). However, therapeutic strategies that promote remyelination or halt disability progression are still lacking (Friese et al., 2014; Singh et al., 2017). Oligodendrocyte precursor cells (OPCs) constitute the main reservoir of myelin-forming oligodendrocytes (OLs) in the CNS, accounting for 5–8% of the glial cells in the adult brain. Under pathological conditions, OPCs possess the intrinsic capacity to differentiate into mature OLs and remyelinate denuded axons, thereby partially restoring neural circuitries and ameliorating functional deficits (Ma et al., 2024). Nevertheless, within a toxic neuroinflammatory microenvironment, remyelination is frequently incomplete and ultimately insufficient to fully compensate myelin loss. Impaired or arrested differentiation of OPCs into mature myelinating OLs is widely recognized as a blockage to effective remyelination. It is also considered as a pathogenic driver of the inexorable progression toward irreversible neurological disability (Ghorbani and Yong, 2021; Gharagozloo et al., 2022).

The ultimate fate commitment of OPCs within the injured areas is governed by local microenvironmental cues (Ghorbani and Yong, 2021), and the regulatory mechanisms underlying this process have been explored for decades. Prior evidence indicates that OPCs can generate not only mature OLs (Yi et al., 2023) but also, under certain conditions, astrocytes (Hu et al., 2010), Schwann cells (Mi et al., 2019), and even neuronal lineages (Heinrich et al., 2014). This implies that insufficient OPCs differentiation toward the OLs, coupled with increased alternative phenotypic transition, may represent a pivotal mechanism leading to remyelination failure. The MS lesion microenvironment is highly complex and characterized by aberrant regulation of signaling cascades that orchestrate OPC development and myelination, including Shh, Notch, bone morphogenic protein (BMP), and Wnt cascades, along with their downstream transcriptional factors, such as Olig1/2, Hes1/5, Smads, and TCFs (Emery, 2010). Notably, elevated fibrinogen levels intrinsic to the MS microenvironment promote BMP pathway activation by upregulating Smad1/5 phosphorylation. This inhibits OPC differentiation into myelinating OLs, while simultaneously driving OPCs to acquire astrocytic characteristics both *in vivo* and *in vitro* (Hu et al., 2010; Petersen et al., 2017). These observations underscore a critical role of BMP signaling in OPC phenotypic transition under MS pathology. Concurrently, previous work from our laboratory and others has demonstrated that excessive activation of Wnt signaling within MS demyelinating lesions impedes OPC differentiation and remyelination (Niu et al., 2021). However, the crosstalk between Wnt and BMP signaling pathways within this pathological microenvironment remains largely unexplored. In particular, how their integrated signals coordinately shape OPC phenotypic transition in the context of MS is still unclear. Elucidating this interplay holds substantial promise for identifying therapeutic targets aimed at restoring remyelination.

In this study, using OPC-tracing transgenic mice, we demonstrate that OPCs acquire astrocytic characteristics in a Lysophosphatidylcholine (LPC)-induced demyelination model. We also uncover a previously unrecognized regulatory paradigm in which opposing BMP and Wnt signals in OPCs antagonistically control phenotypic transition and myelin repair. Strikingly, by bidirectional manipulating both pathways in this model, we further show that targeted suppression of BMP and Wnt signaling prevents pathological astrocyte-like phenotypic transition and promotes robust remyelination. These findings offer mechanistic insights that may guide pro-repair strategies for MS.

## 2 Materials and methods

### 2.1 Mice

The animal experiments were conducted in accordance with protocols approved by the Laboratory Animal Welfare and Ethics Committee of the Third Military Medical University (Army Medical University). All animals were housed under controlled temperature and humidity conditions, with free access to standard diet and water, under a 12-h light/12-h dark cycle. PDGFRα-CreER; Rosa-YFP mice were acquired from Dr. Stephen Fancy at the University of California, San Francisco. The C57BL/6 mice were obtained from Chongqing Lepite Biotechnology Co., Ltd.

### 2.2 Mouse model of demyelination

The preparation of the LPC-induced demyelinating mouse model has been detailed in our previous studies (Niu et al., 2019; Wang et al., 2021). Briefly, mice were anesthetized with isoflurane and placed in a stereotaxic frame. Following scalp incision and skull exposure, 1.5 μL of 1% LPC solution (Sigma-Aldrich, #L0906) was stereotactically injected into the corpus callosum (the injection site was located 1.04 mm lateral and 1.0 mm posterior to bregma, with a depth of 1.62 mm) at a rate of 0.3 μL/min. The needle was left in place for 5 min before withdrawal, after which the scalp incision was sutured. At 1–2 weeks post-injection, brain tissues were collected by transcardial perfusion for subsequent analysis.

### 2.3 Pharmacological interventions

CHIR99021 (Sigma-Aldrich, #SML1046) is an ATP-competitive GSK-3 inhibitor that activates the canonical Wnt signaling pathway. Surgical procedures were performed under general anesthesia induced by isoflurane (1.5–4% in oxygen). Following deep anesthesia, carprofen (5 mg/kg) was administered intraperitoneally for analgesia. Mice were secured in a stereotaxic frame, and a cannula guide was implanted into the lateral ventricle (the injection site was located 1.0 mm lateral and 0.25 mm posterior to bregma, with a depth of 2.5 mm). The cannula pedestal was affixed to the skull with dental cement, reinforced by anchor screws, and sealed with an obturator. The stereotaxic frame was interfaced with a micro-injector, mice received daily intraventricular injections of 2 μL CHIR99021 solution (1.5 μM in 7.5% Dimethyl sulfoxide (DMSO) and 92.5% saline) over a 5-min period for 4 consecutive days (Pachenari et al., 2017). The injection needle was retained for 1-min post-infusion to prevent reflux.

SB4 (MCE, #HY-124697), a potent BMP signaling pathway activator, was dissolved in vehicle (10% DMSO + 90% corn oil) at 10 mg/mL and administered by intraperitoneal injection to conscious mice at 20 mg/kg/day for 7 consecutive days (Xu et al., 2024).

Noggin (R&D Systems, #6057-NG), a potent BMP signaling antagonist, was delivered intracerebroventricularly via an Alzet osmotic pump (Model 1002). Surgical procedures were performed under general anesthesia induced by isoflurane (1.5–4% in oxygen). Following deep anesthesia, carprofen (5 mg/kg) was administered intraperitoneally for analgesia. Mice were secured in a stereotaxic frame, and an Alzet osmotic pump preloaded with Noggin solution was implanted for intracerebroventricular delivery (Meyers et al., 2016). The pump delivered Noggin continuously at 50 ng/μL into the lateral ventricle at a flow rate of 0.25 μL/h for 7 days.

XAV939 (Sigma-Aldrich, #X3004), a potent Wnt signaling pathway antagonist, was dissolved in vehicle (3% DMSO (v/v) in 1% sodium carboxymethylcellulose/phosphate-buffered saline (PBS)) at 2.5 mg/mL and administered by intraperitoneal injection at 10 mg/kg/day for 7 consecutive days.

Due to differences in the dissolution profiles and administration routes of the various drugs, the control group in this study received only an LPC injection. To exclude any potential effects of the drug solvents, three additional experiments were performed: (1) LPC only; (2) LPC with 7.5% DMSO + 92.5% saline (administered via intraventricular injection); and (3) LPC with 10% DMSO + 90% corn oil (administered via intraperitoneal injection). The data confirmed that there were no significant differences in the MBP^+^ area or the number of SOX9^+^ cells within the lesioned area among the three experimental groups (Supplementary Figure S1A). Therefore, LPC injection alone can be directly used as the control.

### 2.4 Immunofluorescent staining

Following deep anesthesia, experimental animals were transcardially perfused with ice-cold PBS, followed by 4% paraformaldehyde (PFA). Brains were harvested and post-fixed in 4% PFA overnight at 4 °C. The fixed brains were then cryoprotected in 30% sucrose solution at 4 °C until fully saturated, embedded in optimal cutting temperature (OCT) compound, and cut into 16-μm-thick coronal sections using a cryostat. Sections were permeabilized with 0.5% Triton X-100 in PBS for 1 h at room temperature (RT), blocked with 2% bovine serum albumin (BSA) for 2 h at RT, and then incubated with primary antibodies diluted in blocking solution overnight at 4 °C. After washing, the sections were labeled with species-matched secondary antibodies for 2 h at RT. The primary antibodies used were as follows: Guinea pig anti-Olig2 (1:500, Oasis Biofarm, #OB-PGP040); Rat anti-EGFP (1:200, Oasis Biofarm, #OB-PRT018); Rat anti-MBP (1:500, Millipore, #MAB395); Goat anti-GFAP (1:500, Abcam, #ab53554); Rabbit anti-SOX9 (1:200, Thermo Fisher Scientific, #702016); Goat anti-SOX9 (1:200, R&D Systems, #AF3075); Rabbit anti-phospho-GSK-3β (Ser9) (1:200, Cell Signaling Technology, #5558); Rabbit anti-SMAD7 (1:200, Abcam, #ab272928); Mouse anti-β-catenin (1:200, BD Biosciences, #610154); Rabbit anti-Phospho-SMAD1/5 (Ser463/465) (1:200, Cell Signaling Technology, #9516); Rat anti-Aldh1l1 (1:200, Oasis Biofarm, #OB-PRT023); Guinea pig anti-EGFP (1:200, Oasis Biofarm, #OB-PGP003); Rabbit anti-CC1 (1:200, Oasis Biofarm, #OB-PRB070); Rat anti-ASPA (1:200, Oasis Biofarm, #OB-PRT005). DAPI was used to counterstain nuclei.

Fluorescent images were captured using either a spinning-disk confocal super-resolution microscope (SpinSR10, Olympus) or a fluorescence microscope (VS200, Olympus). Confocal Z-stack images of brain tissue sections were acquired at 0.5-μm z-step intervals (15 optical sections total) to assess co-localization. For three-dimensional reconstruction, imaging was performed on an Olympus SpinSR10 confocal microscope equipped with a 60 × oil-immersion objective (NA 1.42), with identical parameters applied across all experimental groups. Three-dimensional morphological rendering of oligodendrocytes, astrocytes, and corresponding protein signals was performed using Imaris x64 software (Bitplane, USA). To minimize bias, images were captured by one investigator and then counted by another who was blinded to group allocation. The intensity of nuclear β-catenin, p-Smad1/5, p-Ser9-GSK-3β and Smad7 was measured using ImageJ on confocal images, with the nuclear region defined by DAPI counterstaining.

### 2.5 Transmission electron microscopy

Following anesthesia, animals underwent cardiac perfusion with 0.1 M phosphate buffer, followed by electron microscopy fixative. Subsequently, brain tissues were harvested, sectioned into 1-mm-thick coronal slices, and post-fixed in the same fixative at 4 °C for ≥24 h. Tissue blocks encompassing the injured corpus callosum were carefully dissected for further fixation. After fixation, blocks were rinsed with electron microscopy washing buffer, post-fixed in 1% osmium tetroxide, and serially dehydrated in 50 and 70% acetone. Samples were stained with uranyl acetate dissolved in acetone, and embedded in resin using propylene oxide. After resin polymerization, blocks were trimmed; semi-thin sections were generated to localize lesion regions. Once target areas were identified, ultrathin sections (60 nm) were cut with an ultramicrotome and counter-stained with lead citrate. The corpus callosum was imaged by transmission electron microscopy at magnifications of 2000 × and 4,000×, and acquired micrographs were subjected to quantitative analysis.

### 2.6 Statistics

Statistical analyses were performed using GraphPad Prism software. Normality of data was first assessed using the Shapiro–Wilk test. For comparisons between two groups, an unpaired t-test was applied when the data followed a normal distribution; otherwise, the Mann–Whitney test was used. For comparisons among three groups, a one-way analysis of variance (ANOVA) was performed when the data followed a normal distribution, with Tukey’s multiple comparisons test used for pairwise comparisons. When the data did not follow a normal distribution, the Kruskal-Wallis test was employed, followed by Dunn’s multiple-comparisons test for pairwise comparisons, unless otherwise specified. Statistical analyses of β-catenin, p-Smad1/5, p-Ser9-GSK-3β, and Smad7, as well as the g-ratio obtained from electron microscopy, were performed using a nested mixed-effects model. A probability of *p* < 0.05 was considered to be statistically significant. Significant statistical results are indicated as: *p < 0.05, **p < 0.01, ***p < 0.001, ****p < 0.0001. Data in the main text and figures are presented as mean ± SEM. Throughout the manuscript, the text describes percentage changes relative to the control group, whereas the graphs present fold changes normalized to the same control.

## 3 Results

### 3.1 OPCs acquire astrocytic characteristics following demyelinating injury

To investigate how OPCs respond to injury and how their phenotypic transition affects remyelination, we utilized PDGFRα-CreER; Rosa-YFP transgenic mice in combination with an LPC-induced demyelination model. In these mice, tamoxifen administration drives expression of yellow fluorescent protein (YFP) specifically in PDGFRα^+^ cells, enabling precise labeling and fate tracking of OPCs following injury (Figure 1A)(Wang et al., 2025b). Focal demyelination was induced by injecting LPC into the corpus callosum, allowing assessment of remyelination-related interventions (Figure 1B). We administered tamoxifen to 8-week-old PDGFRα-CreER; Rosa-YFP mice, performed LPC injection at 9 weeks, and collected tissues at 10 weeks. Firstly, YFP immunostaining revealed a marked increase in the number of YFP^+^ cells within the lesion after LPC injection (by 71.13 ± 10.28%) (Figure 1C), indicating substantial accumulation of OPC-derived cells. Moreover, co-immunostaining for YFP and SOX9 (a marker of astrocytes, nearly all SOX9-positive cells within the lesion area were co-labeled with Aldh1l1, a well-established marker of astrocytes, Supplementary Figure S1B) showed that the proportion of YFP^+^/SOX9^+^ cells among YFP^+^ cells increased significantly (by 48.77 ± 9.60%) (Figure 1C), suggesting OPCs acquire astrocytic characteristics following injury. Triple labeling for Aldh1l1, SOX9, and YFP with subsequent three-dimensional reconstruct further substantiated the astrocyte-like phenotypic transition of OPCs (Figure 1D). In addition, we confirmed these findings in C57BL/6 mice, who received LPC at 8 weeks and were harvested for analysis at 10 weeks (Figure 1E). To further confirm the phenotypic transition conclusion, we alternated between SOX9 and GFAP (GFAP, an astrocytic marker in lesion area) as astrocytic markers, as both are widely accepted in the field and provide complementary evidence. Immunostaining for Olig2 (a pan-oligodendroglial lineage marker) demonstrated a significant increase in Olig2^+^ cells in the lesion compared to controls (by 56.70 ± 6.23%) (Figure 1F), reflecting expansion of the oligodendrocyte lineage. Furthermore, the proportion of Olig2^+^/GFAP^+^ cells among Olig2^+^ cells was significantly higher in the LPC model (by 27.96 ± 5.70%) (Figure 1F), indicating enhanced astrocyte-like phenotypic transition of oligodendroglial lineage cells within the lesioned area, which was further validated by quadruple staining for Aldh1l1, Olig2, SOX9, and DAPI, along with subsequent three-dimensional reconstruct (Figure 1G).

**Figure 1:**
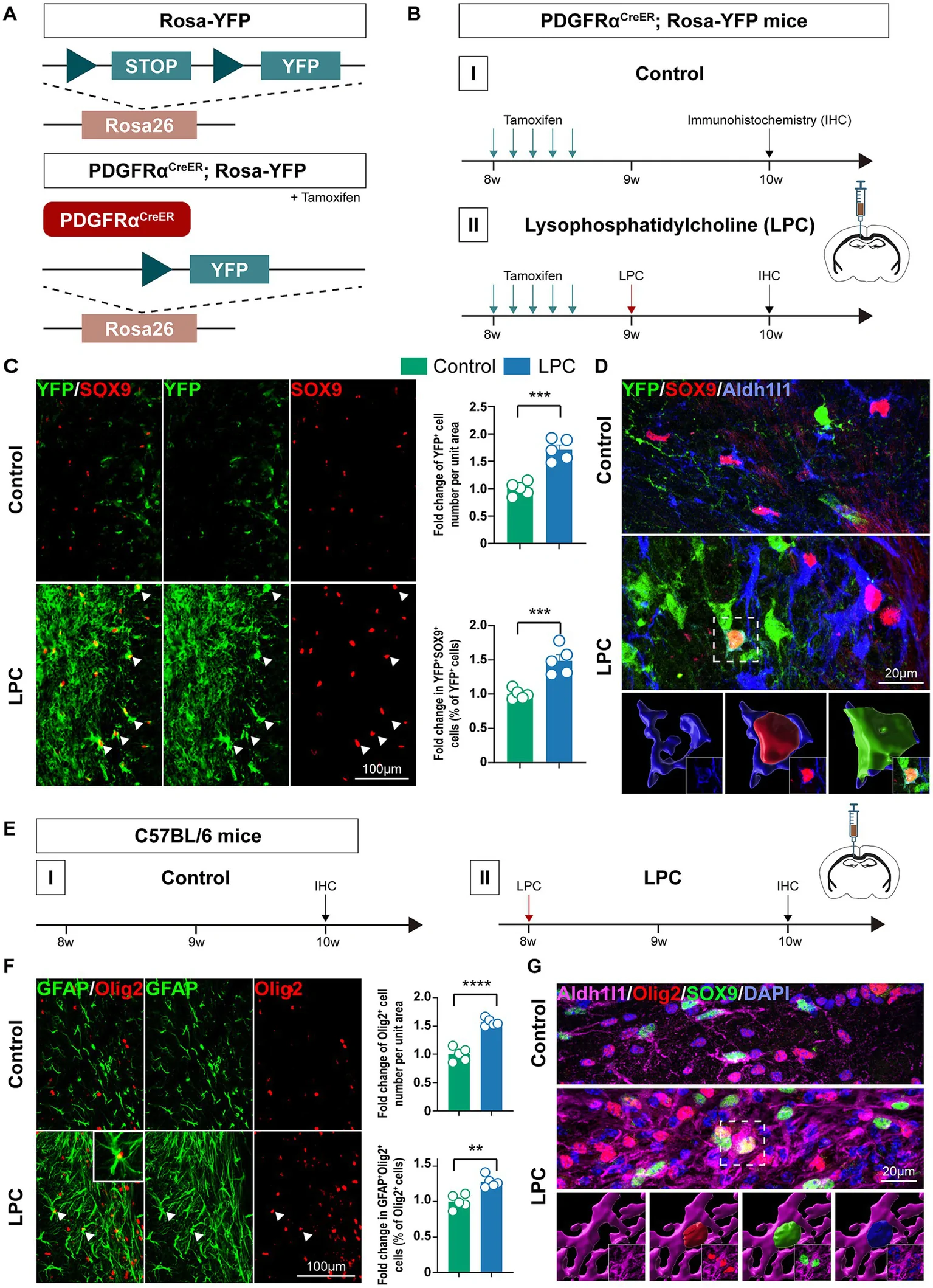
OPCs acquire astrocytic characteristics following demyelinating injury. **(A)** Schematic diagram of the transgene construct in PDGFRα-CreER; Rosa-YFP mice. **(B)** Experimental paradigm for demyelination induction in PDGFRα-CreER; Rosa-YFP mice. **(C)** Representative immunofluorescence images and quantitative analysis of YFP and SOX9 immunostaining in the demyelinated corpus callosum, scale bar = 100 μm, *n* = 5 mice for each group. **(D)** Representative immunofluorescence images and the three-dimensional reconstruction of YFP, SOX9, and Aldh1l1 immunostaining in the demyelinated corpus callosum, scale bar = 20 μm. **(E)** Experimental paradigm for demyelination induction via a single stereotaxic LPC injection into corpus callosum in C57BL/6 mice. **(F)** Representative immunofluorescence images and quantitative analysis of GFAP and Olig2 staining in the demyelinated corpus callosum, scale bar = 100 μm, *n* = 5 mice for each group. **(G)** Representative immunofluorescence images and the three-dimensional reconstruction of Aldh1l1, Olig2, SOX9, and DAPI immunostaining in the demyelinated corpus callosum, scale bar = 20 μm. Intergroup differences were assessed using an unpaired t test. Data were presented as mean ± SEM. **p < 0.01, ***p < 0.001, ****p < 0.0001.

Collectively, these results indicate that during demyelination, OPCs within the lesion undergo a phenotypic transition toward astrocytes.

### 3.2 Opposing activation of BMP and Wnt pathways in transforming OPCs within demyelinating lesions

BMP and Wnt signaling pathways have been established as critical regulators of OPC biology (Cheng et al., 2007; Wang et al., 2025a), governing key processes such as proliferation, differentiation, and phenotypic transition. To explore whether the transformation of OPCs in the LPC-induced demyelination model is associated with alterations in these pathways, we conducted immunostaining to assess the nuclear co-localization of β-catenin (canonical Wnt/β-catenin signaling marker) and phosphorylated glycogen synthase kinase-3 beta at serine 9 (p-Ser9-GSK-3β), which represents the inactivated form of this inhibitor, therefore serving as an indicator of enhanced Wnt signaling (Lv et al., 2020). In parallel, we examined phosphorylated Smad1/5 (p-Smad1/5, positive marker of BMP signaling) and Smad7, an intracellular negative regulator of BMP signaling, whose expression is suppressed upon BMP pathway activation, thus serving as a readout of enhanced BMP signaling (Murakami et al., 2003). In this experiment, PDGFRα-CreER; Rosa-YFP mice (Figure 1B, II) and C57BL/6 mice (Figure 1E, II) were also used for verification. In PDGFRα-CreER; Rosa-YFP mice, compared to YFP^+^ single-positive cells, nuclear β-catenin levels in Aldh1l1^+^/YFP^+^ double-positive cells were found to be significantly lower, by 46.87 ± 19.03%, indicative of pronounced downregulation of Wnt signaling in OPC-derived astrocyte-like cells (Figure 2A). Moreover, relative to YFP^+^ single-positive cells, nuclear p-Smad1/5 levels were markedly elevated in Aldh1l1^+^/YFP^+^ double-positive cells (130.7 ± 56.14%), corroborating that BMP signaling was robustly enhanced in the transforming OPCs (Figure 2A). Similarly, in C57BL/6 mice, our results revealed that p-Ser9-GSK-3β levels were significantly lower in GFAP^+^/Olig2^+^ double-positive cells (which representing OPC-derived astrocyte-like cells undergoing transformation) than in Olig2^+^ single-positive cells (representing untransformed or less transformed OPCs), showing a reduction of 56.96 ± 23.45% (Figure 2B). This decrease indicates a marked decrease of Wnt signaling activity in the transforming cells. In contrast, Smad7 expression was significantly reduced in GFAP^+^/Olig2^+^ double-positive cells compared with Olig2^+^ cells, with a decrease of 51.00 ± 20.99% (Figure 2B). Given that Smad7 normally functions as a negative feedback inhibitor of BMP receptor signaling, its downregulation reflects increased BMP signaling activity. Collectively, these findings suggest that transforming cells within the lesion area exhibit an aberrant concurrent upregulation of BMP signaling alongside downregulation of Wnt signaling.

**Figure 2:**
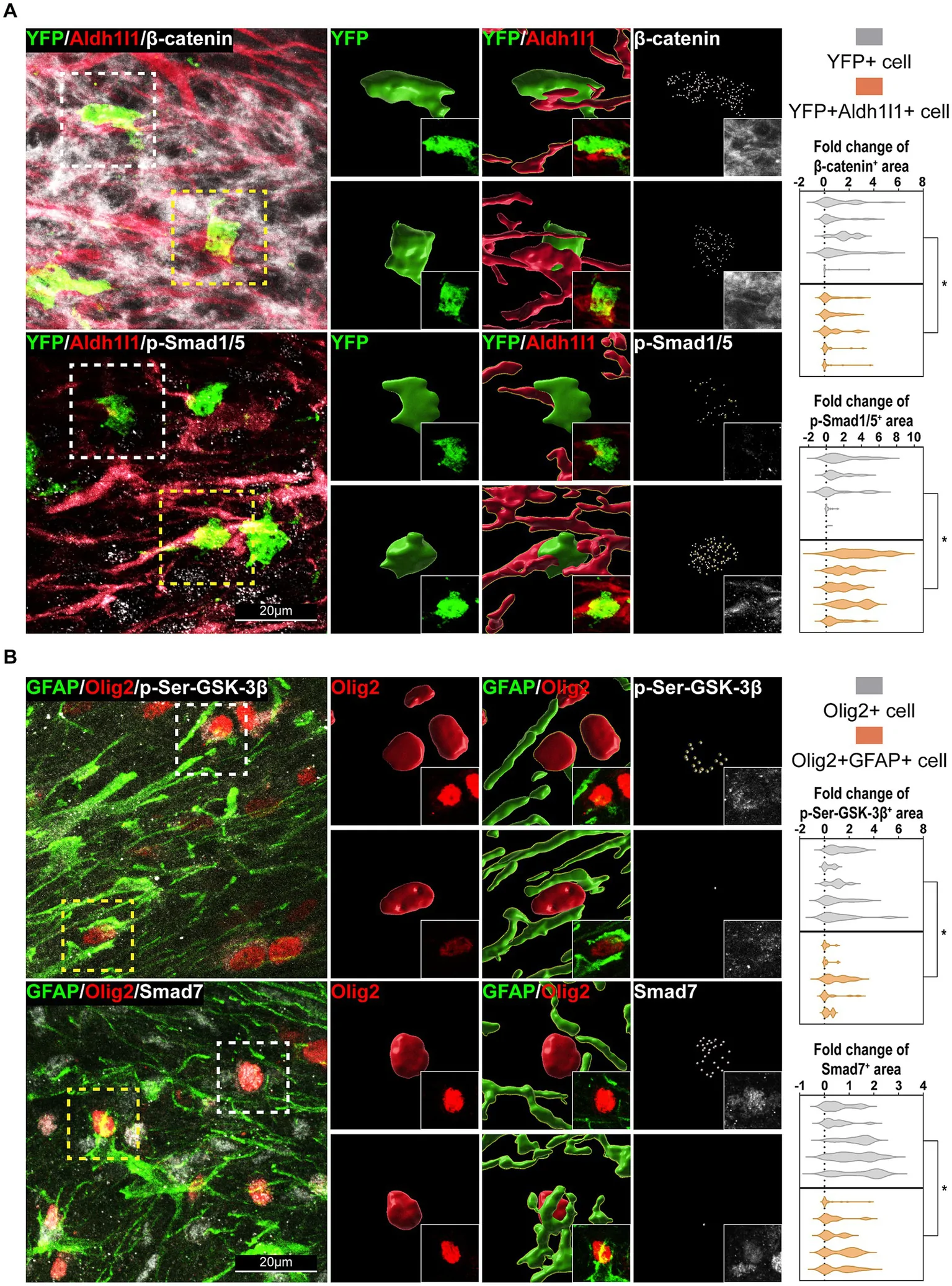
Opposing activation of BMP and Wnt pathways in transforming OPCs within demyelinating lesions. **(A)** Representative immunofluorescence images, the three-dimensional reconstruction, and quantitative analysis of YFP, Aldh1l1, and β-catenin or p-Smad1/5 immunostaining in the individual cells of the demyelinated corpus callosum. Cells within the white dashed box are considered YFP single-positive cells, whereas cells within the yellow dashed box are considered YFP^+^Aldh1l1^+^ double-positive cells. Scale bar = 20 μm. **(B)** Representative immunofluorescence images, the three-dimensional reconstruction, and quantitative analysis of Olig2, GFAP, and p-Ser-GSK-3β or Smad7 immunostaining in the individual cells of the demyelinated corpus callosum. Cells within the white dashed box are considered Olig2 single-positive cells, whereas cells within the yellow dashed box are considered GFAP^+^Olig2^+^ double-positive cells. Scale bar = 20 μm. In all experiments, each group contained 5 animals, and at least 50 cells were recorded, with an average of more than 10 cells per animal. Intergroup differences were assessed using a nested t test. *p < 0.05.

These opposing changes in pathway activations may synergistically drive the phenotypic transition of OPCs toward astrocytes; however, whether they cooperate or antagonize each other in disease remains unclear.

### 3.3 Aligned single modulation of BMP or Wnt signaling does not affect OPC phenotypic transition or remyelination

To investigate the dynamic interplay between BMP and Wnt signaling during remyelination, we examined their roles in OPC phenotypic transition and the remyelination process. Instead of opposing endogenous signaling trends, our experimental strategies were designed to align with the natural trajectory of these pathways. Operationally, we pharmacologically modulated the LPC-induced focal demyelination model by either activating BMP signaling with SB4, a canonical BMP pathway activator (Xu et al., 2024) or inhibiting Wnt signaling with XAV939, a Wnt inhibitor (Yin et al., 2024). C57BL/6 mice received a single stereotaxic LPC injection at 8 weeks of age. At a one-week post-lesion time point, when endogenous OPCs are actively proliferating and beginning to differentiate (Niu et al., 2019), mice were administered SB4 or XAV939. Tissues were then collected one week after administration (Figure 3A). All compounds were administered continuously for seven consecutive days, and tissues were collected at the experimental endpoint. However, subsequent immunostaining for phenotypic transition and myelin markers (Figures 3B–D) revealed that, following LPC injection, neither further upregulation of BMP signaling nor downregulation of Wnt signaling affected the number of SOX9^+^ cells or altered the counts of Olig2^+^ cells and Aldh1l1^+^/Olig2^+^ double-positive cells (Figures 3B,D), suggesting that pharmacological activation of BMP or inhibition of Wnt alone cannot drive OPCs toward astrocytic phenotype. In parallel, we assessed changes in mature oligodendrocyte markers (CC1 and ASPA) and myelin basic protein (MBP) within remyelinating areas, and observed no evidence of differentiation arrest or additional impairment of myelin deposition (Figures 3B,C). Further validation using transmission electron microscopy corroborated that neither activation of BMP nor inhibition of Wnt had an effect on myelin ultrastructure (Figure 3E).

**Figure 3:**
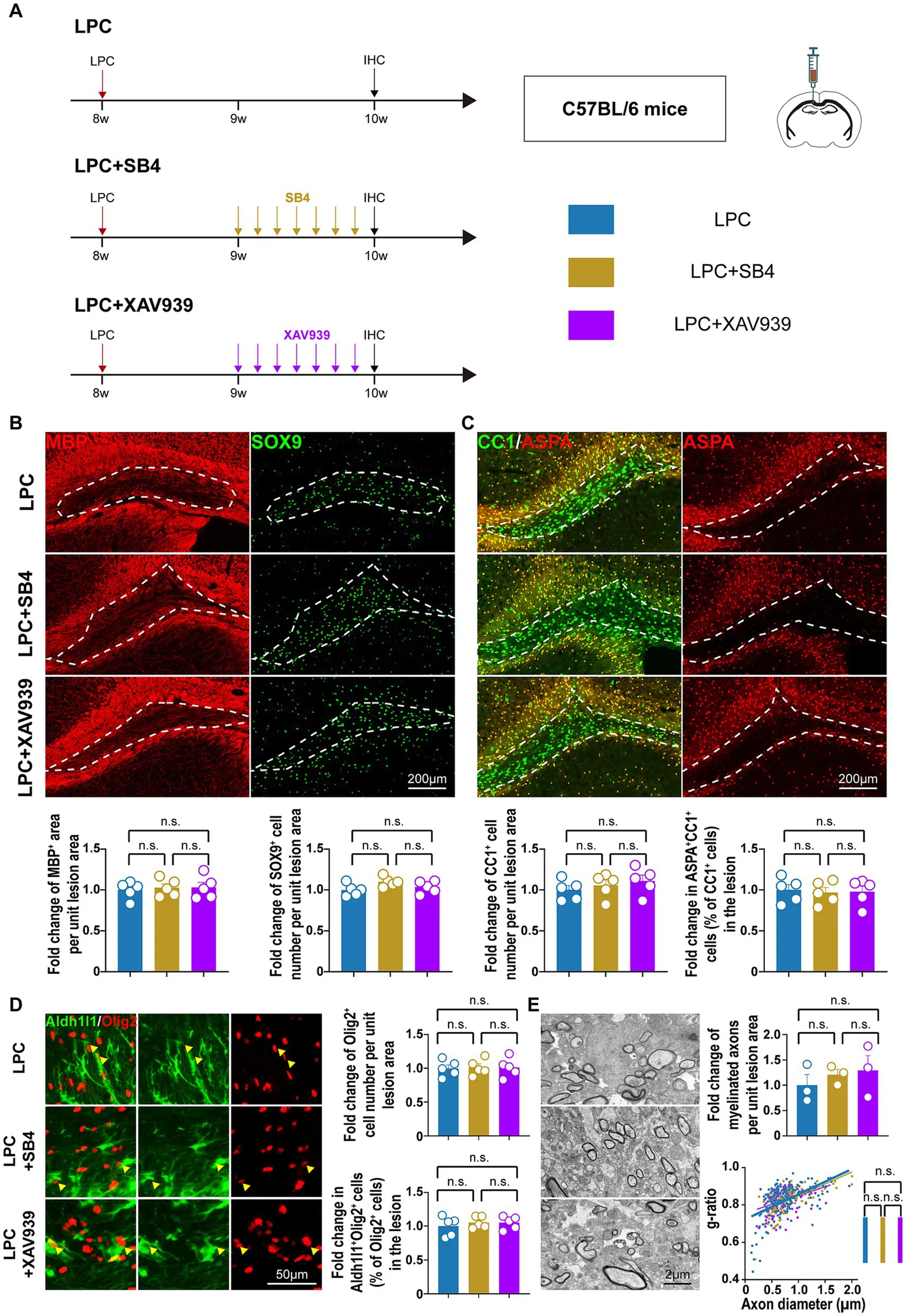
Aligned single modulation of BMP or Wnt signaling does not affect OPC phenotypic transition or remyelination. **(A)** Experimental paradigm for LPC-induced demyelination in C57BL/6 mice and therapeutic interventions with SB4 or XAV939 administration. **(B)** Representative immunofluorescence images and quantitative analysis of MBP and SOX9 immunostaining in the demyelinated corpus callosum, scale bar = 200 μm, *n* = 5 mice for each group. **(C)** Representative immunofluorescence images and quantitative analysis of CC1 and ASPA immunostaining in the demyelinated corpus callosum, scale bar = 200 μm, *n* = 5 mice for each group. **(D)** Representative immunofluorescence images and quantitative analysis of Aldh1l1 and Olig2 immunostaining in the demyelinated corpus callosum, scale bar = 50 μm, *n* = 5 mice for each group. **(E)** Electron microscopy images, the number of myelinated axons and g-ratio calculations of myelinated axons in the demyelinated corpus callosum, scale bar = 2 μm, at least 3 mice for each group. Intergroup comparisons of the g-ratio were analyzed using a nested one-way ANOVA with Tukey’s multiple comparisons test, whereas all other intergroup comparisons were performed using one-way ANOVA followed by Tukey’s multiple comparisons test. Data were presented as mean ± SEM.

Given that enhanced BMP signaling or suppressed Wnt signaling do not further aggravate OPC phenotypic transition or impair remyelination, this evidence suggests that opposing changes in BMP and Wnt signaling primarily act antagonistically, rather than cooperatively in the same regulatory direction.

### 3.4 Isolating BMP and Wnt signaling reveals opposing contributions to OPC phenotypic transition and myelin repair

To further dissect the distinct contributions of BMP and Wnt signaling to OPC phenotypic transition and remyelination in disease, we established a strategy to isolate the effect of each pathway by eliminating the activity of one while preserving the activity of the other. To implement this strategy operationally, C57BL/6 mice received LPC injection at 8 weeks of age, followed by treatment with Noggin, an inhibitor of BMP signaling (Meyers et al., 2016), or CHIR99021, a selective Wnt/β-catenin pathway activator (Pachenari et al., 2017) (Figure 4A). We next evaluated OPC transformation and remyelination by immunostaining and transmission electron microscopy. Compared with the control group, mice treated with Noggin alone or CHIR99021 alone exhibited significant alternations in OPC phenotypic transition and the remyelination process (Figures 4B–E). Normalized to the control group, mice treated with Noggin alone exhibited a significantly increase in MBP^+^ area by 27.75 ± 5.66% and a marked reduction in SOX9^+^ cell count by 41.62 ± 7.84% (Figure 4B). Although the number of CC1^+^ cells remained unchanged, the proportion of cells co-expressing ASPA and CC1 was significantly elevated (35.51 ± 5.18%) (Figure 4C). Furthermore, electron microscopy confirmed a substantial increase in myelinated axons (157.7 ± 27.67%) within the lesion area (Figure 4E). Meanwhile, Olig2^+^ cell counts in the lesioned region remained unaltered, whereas Aldh1l1^+^/Olig2^+^ double-positive cells were significantly decreased (33.33% ± 5.15%) (Figure 4D). Collectively, these findings indicate that, in our model, eliminating BMP signaling is sufficient to promote remyelination and suppress astrocyte-like phenotypic transition of OPCs, suggesting that BMP upregulation is deleterious. Given the antagonistic relationship between Wnt and BMP pathways, this observation indirectly supports a beneficial role of downregulated Wnt signaling in remyelination and OPC differentiation. In sharp contrast, CHIR99021 monotherapy not only increased the number of SOX9^+^ cells (66.76% ± 18.55%) and Aldh1l1^+^/Olig2^+^ double-positive cells (33.08% ± 5.46%), but also significantly decreased MBP^+^ area (17.54% ± 5.55%), CC1^+^ cell counts (31.21% ± 6.14%), and myelinated axons number (71.35% ± 6.28%) relative to control (Figures 4B–E), suggesting that counteracting the endogenous Wnt downregulation drove the disease toward a worse outcome. In light of the antagonistic relationship between Wnt and BMP pathways, this result provides complementary evidence that upregulated BMP signaling exerts a detrimental effect on remyelination and OPC differentiation.

**Figure 4:**
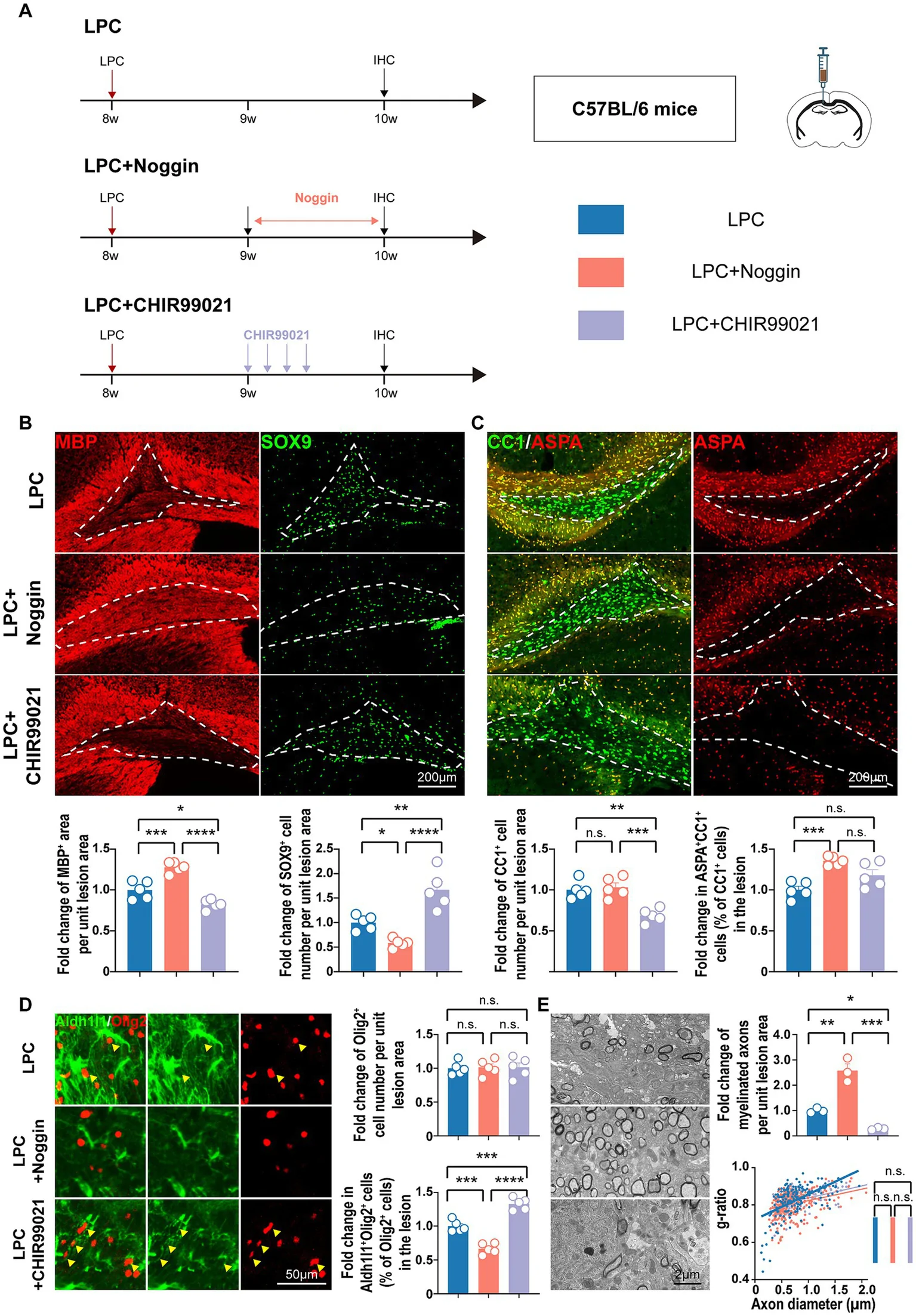
Isolating BMP and Wnt signaling reveals opposing contributions to OPC phenotypic transition and myelin repair. **(A)** Experimental paradigm for LPC-induced demyelination in C57BL/6 mice and therapeutic interventions with Noggin or CHIR99021 administration. **(B)** Representative immunofluorescence images and quantitative analysis of MBP and SOX9 immunostaining in the demyelinated corpus callosum, scale bar = 200 μm, *n* = 5 mice for each group. **(C)** Representative immunofluorescence images and quantitative analysis of CC1 and ASPA immunostaining in the demyelinated corpus callosum, scale bar = 200 μm, *n* = 5 mice for each group. **(D)** Representative immunofluorescence images and quantitative analysis of Aldh1l1 and Olig2 immunostaining in the demyelinated corpus callosum, scale bar = 50 μm, *n* = 5 mice for each group. **(E)** Electron microscopy images, the number of myelinated axons and g-ratio calculations of myelinated axons in the demyelinated corpus callosum, scale bar = 2 μm, at least 3 mice for each group. Intergroup comparisons of the g-ratio were analyzed using a nested one-way ANOVA with Tukey’s multiple comparisons test, whereas all other intergroup comparisons were performed using one-way ANOVA followed by Tukey’s multiple comparisons test. Data were presented as mean ± SEM. *p < 0.05, **p < 0.01, ***p < 0.001, ****p < 0.0001.

Taken together, these results demonstrated that both BMP and Wnt pathways are critical regulators of OPC phenotypic transition and remyelination: upregulated BMP signaling is deleterious, whereas downregulated Wnt signaling is beneficial. This implies that their aberrant dynamic interplay may pose a barrier to effective myelin repair in demyelinating diseases.

### 3.5 Pharmacological co-inhibition of BMP and Wnt signaling blocks astrocyte-like phenotypic transition and facilitates remyelination

To further advance the translational potential of our findings toward future clinical applications, we next investigated whether pharmacological co-downregulation or co-upregulation of these two signaling pathways could, respectively, promote beneficial or detrimental effects on remyelination. Operationally, C57BL/6 mice received LPC injection at 8 weeks of age, followed by the combination of Noggin and XAV939, or the combination of SB4 and CHIR99021; tissues were collected one-week post-administration at the experimental endpoint (Figure 5A).

**Figure 5:**
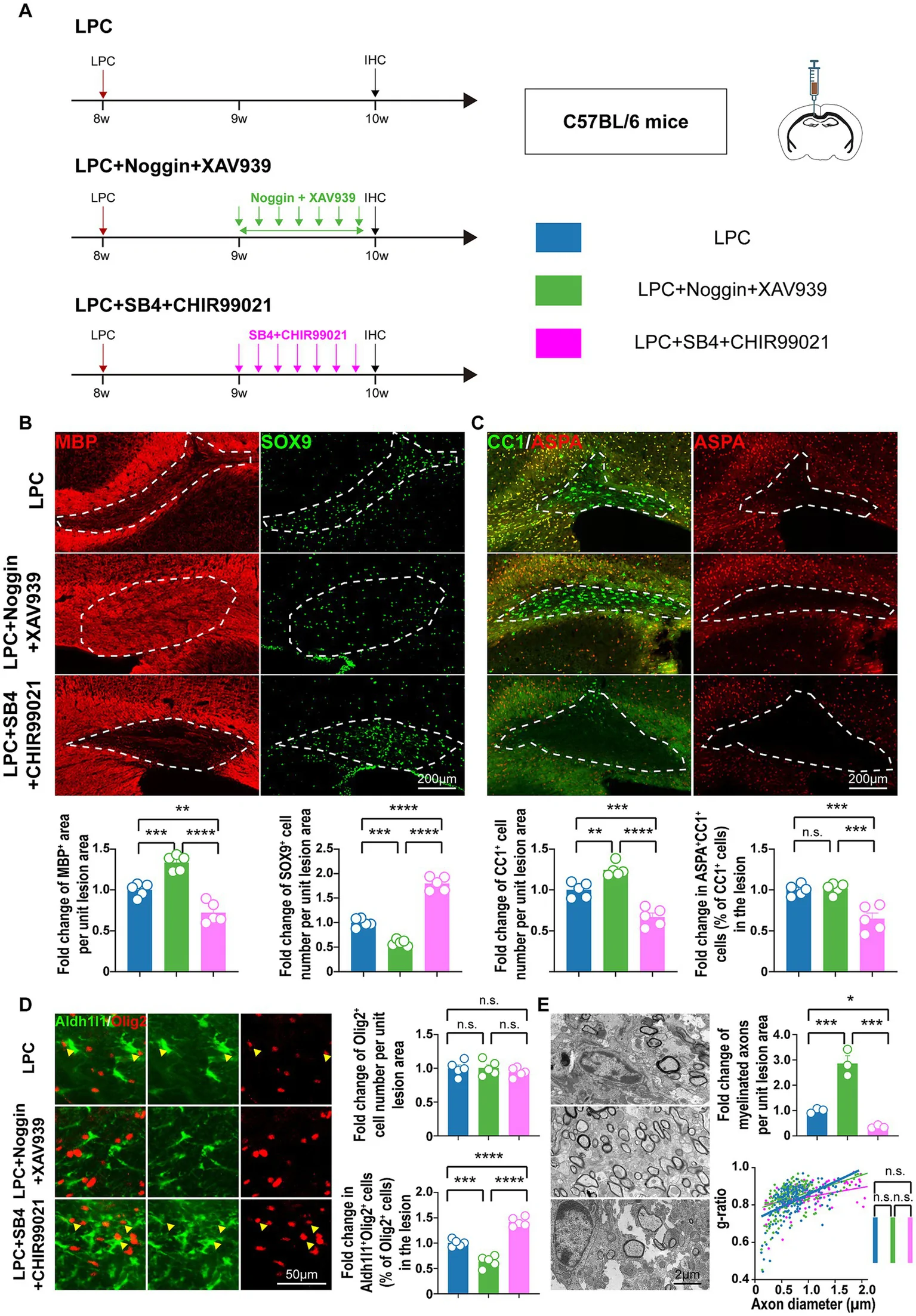
Pharmacological co-inhibition of BMP and Wnt signaling blocks astrocyte-like phenotypic transition and facilitates remyelination. **(A)** Experimental paradigm for LPC-induced demyelination in C57BL/6 mice and therapeutic interventions with Noggin+XAV939 or SB4 + CHIR99021 administration. **(B)** Representative immunofluorescence images and quantitative analysis of MBP and SOX9 immunostaining in the demyelinated corpus callosum, scale bar = 200 μm, *n* = 5 mice for each group. **(C)** Representative immunofluorescence images and quantitative analysis of CC1 and ASPA immunostaining in the demyelinated corpus callosum, scale bar = 200 μm, *n* = 5 mice for each group. **(D)** Representative immunofluorescence images and quantitative analysis of Aldh1l1 and Olig2 immunostaining in the demyelinated corpus callosum, scale bar = 50 μm, *n* = 5 mice for each group. **(E)** Electron microscopy images, the number of myelinated axons and g-ratio calculations of myelinated axons in the demyelinated corpus callosum, scale bar = 2 μm, at least 3 mice for each group. Intergroup comparisons of the g-ratio were analyzed using a nested one-way ANOVA with Tukey’s multiple comparisons test, whereas the number of myelinated axons was performed using one-way ANOVA with Holm-Šídák’s *post hoc* test, and all other intergroup comparisons were performed using one-way ANOVA with Tukey’s *post hoc* test. Data were presented as mean ± SEM. *p < 0.05, **p < 0.01, ***p < 0.001, ****p < 0.0001.

In our results, immunostaining for phenotypic transition and myelin markers revealed that the co-upregulation of Wnt and BMP signaling using SB4 and CHIR99021 significantly increased the number of SOX9^+^ cells by 79.94 ± 7.51% and Aldh1l1^+^/Olig2^+^ double-positive cells (41.76% ± 5.14%), while concomitantly decreasing MBP^+^ area (27.76% ± 6.80%), CC1^+^ cell counts (33.15% ± 6.52%), and myelinated axon numbers (63.72% ± 6.57%) (Figures 5B–E), suggesting that the concurrent pathway activation potentially skews OPCs toward astrocytic phenotype rather than differentiating into myelinating OLs. This effect is attributable to the fact that SB4 further potentiated the harmful upregulated BMP signaling, whereas CHIR99021 attenuated the beneficial downregulated Wnt signaling. Strikingly, concurrent blockade of both BMP and Wnt signaling with Noggin and XAV939 not only effectively increased MBP^+^ area (33.43% ± 5.62%), CC1^+^ cell numbers (24.46% ± 5.54%), and myelinated axon counts (186.3% ± 30.55%), but also significantly reduced the number of SOX9^+^ cells (40.75% ± 4.90%) and Aldh1l1^+^/Olig2^+^ double-positive cells (36.26% ± 5.92%) (Figures 5B–E). In this setting, Noggin attenuated the harmful upregulated BMP signaling, while XAV939 further enhanced the beneficial downregulated Wnt signaling. Collectively, these results suggest that dual inhibition of the BMP and Wnt pathways may suppress the astrocyte-like phenotypic transition of OPCs, thereby facilitating remyelination.

### 3.6 The beneficial role of Wnt downregulation is balanced by the detrimental role of BMP upregulation

To assess whether the beneficial effect of Wnt downregulation outweighs the harmful effect of BMP upregulation, we treated mice with SB4 + XAV939 (to exacerbate both the beneficial and deleterious effects) or Noggin + CHIR99021 (to eliminate both effects simultaneously). However, compared with controls, neither combination significantly affected OPC phenotypic transition or remyelination, as indicated by comparable MBP^+^ area, SOX9^+^ cell counts, CC1^+^ cell numbers, Aldh1l1^+^/Olig2^+^ double-positive cells, and myelinated axon counts, with no statistically significant changes observed (Figures 6A–E). These findings indicate that, within the lesioned area of the LPC-induced focal demyelination model, the beneficial effect of Wnt downregulation is balanced by the deleterious effect of BMP upregulation.

**Figure 6:**
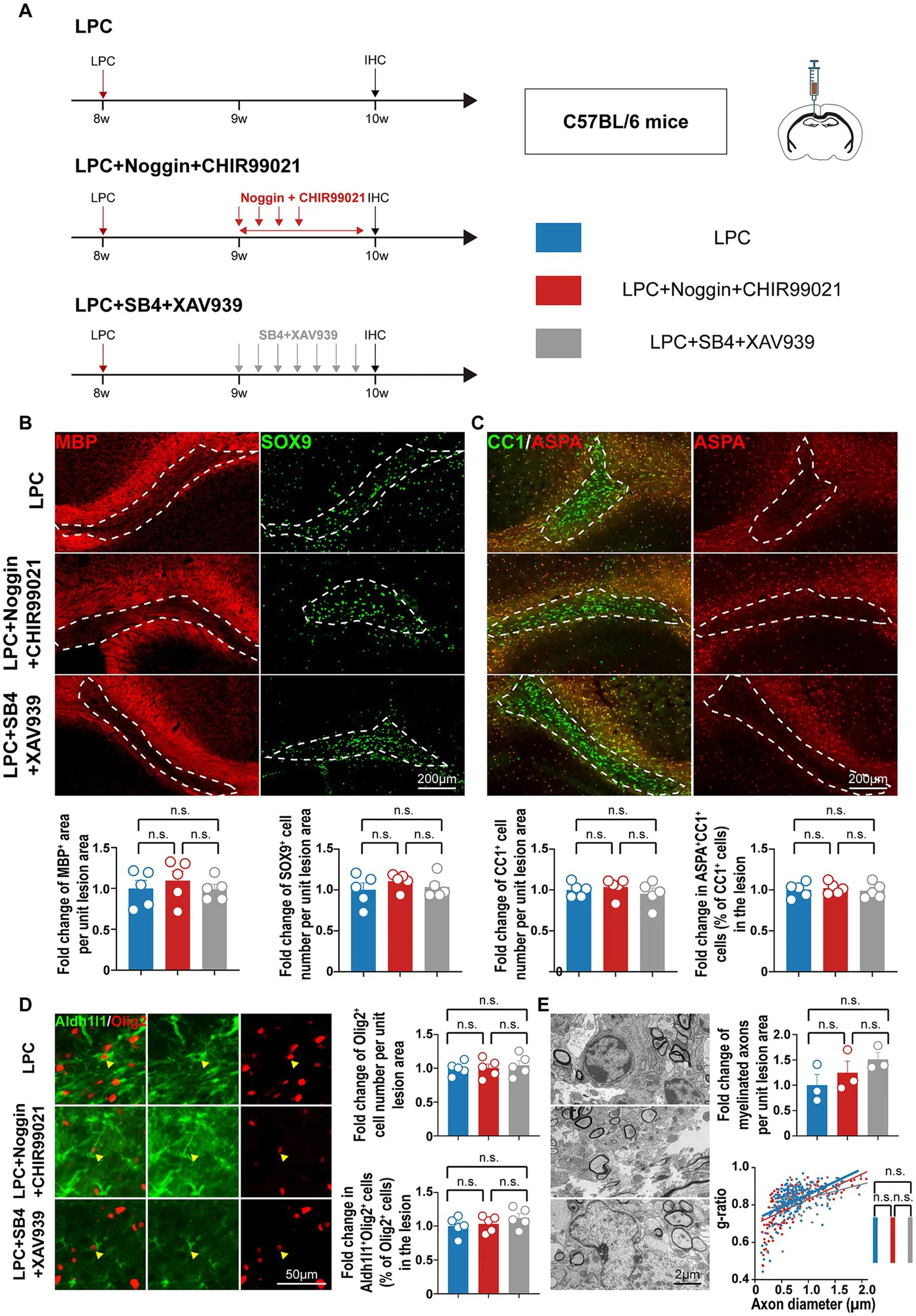
The beneficial role of Wnt downregulation is balanced by the detrimental role of BMP upregulation. **(A)** Experimental paradigm for LPC-induced demyelination in C57BL/6 mice and therapeutic interventions with Noggin+ CHIR99021 or SB4 + XAV939 administration. **(B)** Representative immunofluorescence images and quantitative analysis of MBP and SOX9 immunostaining in the demyelinated corpus callosum, scale bar = 200 μm, *n* = 5 mice for each group. **(C)** Representative immunofluorescence images and quantitative analysis of CC1 and ASPA immunostaining in the demyelinated corpus callosum, scale bar = 200 μm, *n* = 5 mice for each group. **(D)** Representative immunofluorescence images and quantitative analysis of Aldh1l1 and Olig2 immunostaining in the demyelinated corpus callosum, scale bar = 50 μm, *n* = 5 mice for each group. **(E)** Electron microscopy images, the number of myelinated axons and g-ratio calculations of myelinated axons in the demyelinated corpus callosum, scale bar = 2 μm, at least 3 mice for each group. Intergroup comparisons of the g-ratio were analyzed using a nested one-way ANOVA with Tukey’s multiple comparisons test, whereas the number of CC1^+^ cells was performed using the Kruskal-Wallis test with Dunn’s *post hoc* test, and all other intergroup comparisons were performed using one-way ANOVA followed by Tukey’s multiple comparisons test. Data were presented as mean ± SEM.

## 4 Discussion

In this study, we reveal that OPCs acquire astrocytic characteristics, driven by the simultaneous and opposing activation of BMP and Wnt signaling. Through pharmacological bidirectional manipulation of both pathways *in vivo*, we further demonstrate that BMP and Wnt signaling act antagonistically to regulate the balance between OPC differentiation and astrocyte-like phenotypic transition, thereby controlling myelin repair (Figure 7). These findings provide new mechanistic insights for therapeutic strategies in MS.

**Figure 7:**
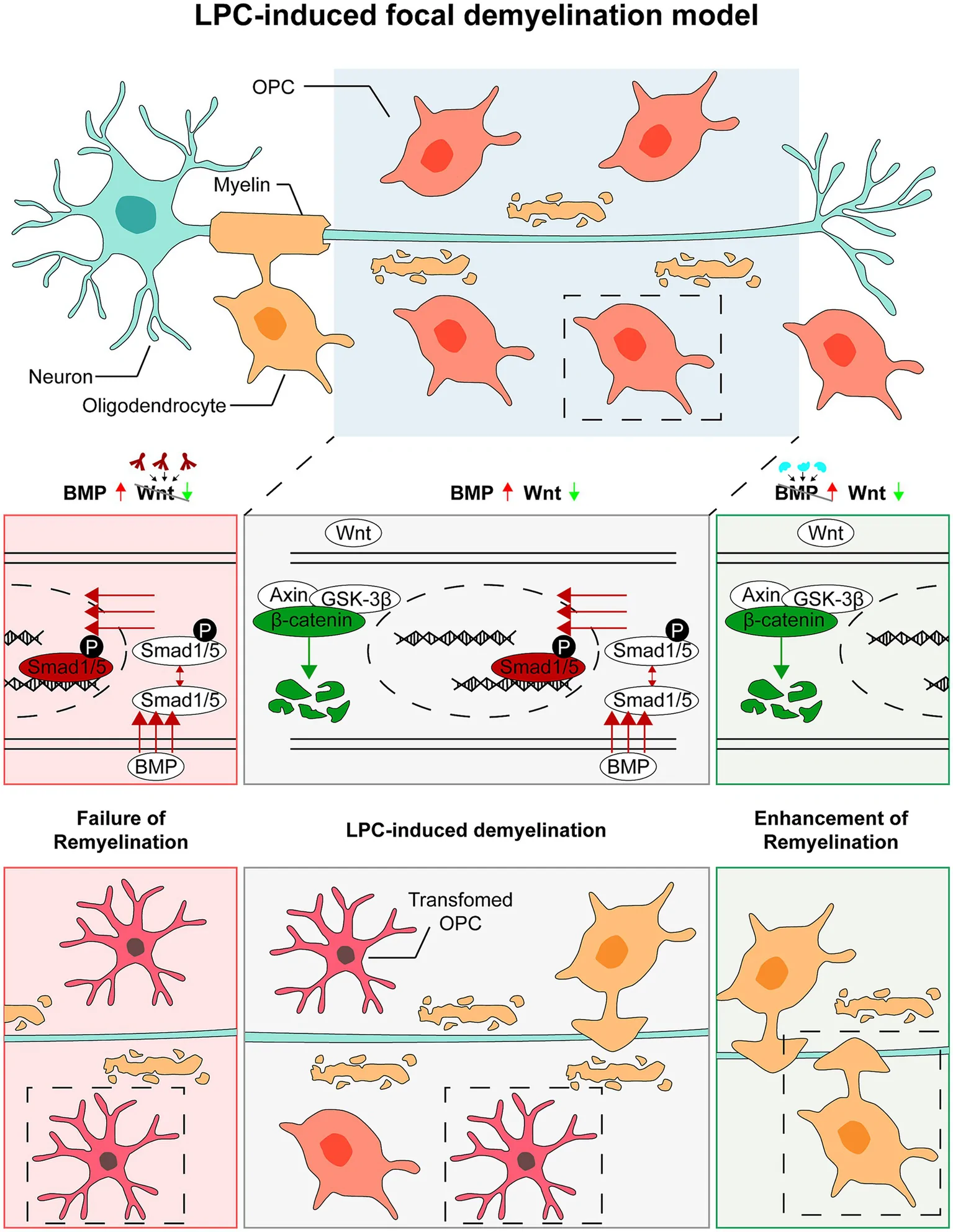
Schematic representation of the mechanism underlying astrocyte-like phenotypic transition of OPCs in the LPC-induced focal demyelination model. The demyelinating microenvironment dictates astrocyte-like phenotypic transition of OPCs by inducing Wnt downregulation and BMP upregulation in transforming OPCs. Given their reciprocal antagonism, Wnt downregulation is protective whereas BMP upregulation is detrimental; thus, pharmacologically counteracting either pathway can influence OPC phenotypic transition and remyelination.

In chronic neurodegenerative diseases such as MS, inflammatory factors in the microenvironment activate OPCs. These cells migrate to and proliferate within lesions (Niu et al., 2019), where they are expected to differentiate and remyelinate, but their reparative capacity is modulated by the glial scar (Adams and Gallo, 2018) that both restricts pathology spread and influences tissue regeneration (Marangon et al., 2024). In the present study, we establish that OPCs within demyelinating lesions undergo phenotypic transition toward astrocytes. This phenotypic transition is not a passive response; rather, we show that transforming cells concurrently exhibit elevated nuclear p-Smad1/5 and reduced Smad7 (indicative of BMP activation) alongside diminished nuclear β-catenin and p-Ser9-GSK-3β (reflective of Wnt suppression). These observations suggest that the net balance between the two pathways determines this pathological phenotypic transition.

Early *in vitro* studies showed OPCs could differentiate into astrocytes (Raff et al., 1983; Kondo and Raff, 2000), which may be due in part to the fact that astrocytes and oligodendrocytes originate from the same lineage (Huang et al., 2021). Subsequent evidence indicates that OPCs retain this capacity even in adulthood, likely reflecting a dysregulated adaptation of OPCs to the altered microenvironment in disease (Hackett et al., 2018; Weng et al., 2019; Kirdajova et al., 2021). Multiple microenvironmental cues have been shown to perturb OPC differentiation and drive their phenotypic transition toward astrocytes (Buchanan and Cheadle, 2025). Although the BMP signaling pathway has been well characterized in OPC phenotypic transition (Mabie et al., 1997; Hu et al., 2010) and canonical Wnt/β-catenin signaling pathway exerts context-dependent effects on oligodendroglial development (Guo et al., 2015), their crosstalk in OPC-to-astrocyte transformation has remained unclear. Previously, *in vitro* evidence suggested that the inhibitory effect of Wnt pathway activation on OPC differentiation requires cooperation with BMP signaling (Feigenson et al., 2011); however, whether such coordinated regulation occurs in the MS lesion microenvironment *in vivo* was unknown. Here, our pharmacological manipulations provide direct causal evidence for an antagonistic relationship between BMP and Wnt pathways. When we aligned with the endogenous trends (activating BMP with SB4 or inhibiting Wnt with XAV939 alone), we observed no effect on OPC phenotypic transition, or myelin parameters, indicating that neither pathway acts independently to drive pathology. In contrast, isolating each pathway by blocking the other (Noggin alone or CHIR99021 alone) produced divergent and significant outcomes, confirming that the opposing changes in BMP and Wnt signaling act antagonistically rather than cooperatively. Strikingly, eliminating BMP signaling while retaining Wnt signaling was sufficient to suppress astrocyte-like phenotypic transition of OPCs and promote remyelination, establishing that upregulated BMP is detrimental whereas downregulated Wnt is beneficial, and that their effects are mutually opposing. This also implies that while normal OPC differentiation into myelinating oligodendrocytes promotes remyelination, their astrocyte-like phenotypic transition represents an alternative mechanism that impedes repair, independent of differentiation arrest.

A growing body of evidence indicates that OPCs are a cell type with multifaceted functional potentials (Butt et al., 2026). Under pathological conditions, they exhibit a spectrum of responses, including the newly generated astrocytes and OPCs migrate into the lesion from surrounding areas, contributing to repair and functional circuit regeneration (Shafqat et al., 2023). Drawing on the concept of “gliosis,” which encompasses complex glial responses in diseases, we propose the term “OPC-osis” to describe this broad repertoire of OPC reactivity (Yi et al., 2023). Compared with other glial responses, OPC-osis holds distinct significance. For example, promoting OPC differentiation with clemastine (Mei et al., 2014) alleviates chronic demyelinating optic neuropathy in relapsing–remitting MS patients (Green et al., 2017). OPCs also can acquire the characteristics of Schwann cells and express the Schwann cell marker S100β (Mi et al., 2019), highlighting the potential for myelin regeneration (Chen et al., 2021). Moreover, SOX2 can induce OPCs to transform into neurons capable of receiving synaptic inputs in injured brain, offering avenues for neuronal repair (Heinrich et al., 2014). However, as reflected by OPCs transforming into astrocytes and contributing to glial scar formation (Hu et al., 2010), uncontrolled OPC differentiation or phenotypic transition often obstacle tissue repair. In this study, we show that it is the integrated, opposing activity of BMP and Wnt, rather than isolated signals, that dictates whether OPCs contribute to remyelination or to glial scar formation. This result-driven framework clarifies why previous single-pathway interventions have often been insufficient and provides a mechanistic rationale for combinatorial strategies. Indeed, pharmacological co-inhibition of BMP and Wnt signaling not only suppressed astrocyte-like phenotypic transition of OPCs but also facilitated remyelination, underscoring that OPC phenotypic transition critically determine the efficiency of myelin repair under pathological conditions.

Our study has several limitations. First, our pharmacological agents were not delivered specifically to OPCs, so off-target effects on microglia, astrocytes, or the blood–brain barrier cannot be excluded. Future studies using transgenic mice that allow OPC-specific knock-in or knock-out of Wnt and BMP signaling components will be needed to provide more definitive evidence. Second, we did not detect significant changes in either the MBP^+^ area or the number of SOX9^+^ cells following treatment with XAV939 or SB4 individually. This lack of effect may be explained by at least two possibilities: (i) the level of Wnt pathway activation may not be as effectively regulated as that of the BMP pathway under our experimental conditions; or (ii) the BMP and Wnt pathways may have an upstream–downstream relationship, wherein one pathway is permissive for the other. Finally, we acknowledge that no single marker can be interpreted as definitive evidence of pathway activity. In this study, we therefore used Wnt and BMP signaling markers in a complementary manner, and this panel of markers provides consistent information supporting our interpretation.

## Data Availability

The raw data supporting the conclusions of this article will be made available by the authors, without undue reservation.

## Supplementary material

SUPPLEMENTARY FIGURE S1 **(A)** MBP and SOX9 staining in the lesion area of the LPC‑induced demyelination model. Representative immunofluorescence images and quantitative analysis of MBP and SOX9 immunostaining in the demyelinated corpus callosum, scale bar = 100µm, *n* = 3 mice for each group. **(B)** Aldh1l1/SOX9 co‑staining in the lesion area of the LPC‑induced demyelination model. Representative immunofluorescence images and quantitative analysis of Aldh1l1 and SOX9 immunostaining in the demyelinated corpus callosum, scale bar = 50µm, *n* = 3 mice for each group. Intergroup comparisons of the MBP+ area and the SOX9+ cell count were analyzed using one‑way ANOVA followed by Tukey’s multiple comparisons test. Data were presented as mean ± SEM.
